# Toward a Functional and Conceptual Framework for Adhesive Materials: The Role of Etching Technique

**DOI:** 10.3390/dj14020119

**Published:** 2026-02-18

**Authors:** Miguel Angel Muñoz, Issis Luque-Martinez

**Affiliations:** 1 Dental Materials Department, Research Center in Dental and Medical Sciences, Faculty of Dentistry, Universidad de Valparaíso, Valparaíso 2360004, Chile; issis.luque@uv.cl; 2 Master’s in Dentistry Sciences Program, Faculty of Dentistry, Universidad de Valparaíso, Valparaíso 2360004, Chile

**Keywords:** etching technique, adhesive system classification, universal adhesives, self-adhesive materials, touch-cure primers, smear layer

## Abstract

**Background/Objectives**: The classification of adhesive systems has historically relied on the type of etching agent and the sequence of application steps, distinguishing etch-and-rinse and self-etch categories. However, these models do not encompass the versatility introduced by universal adhesives or other emerging polymeric materials. This review aimed to integrate etching technique as a defining parameter within adhesive classification, linking material composition, bonding strategy, and clinical execution into a coherent functional framework. **Methods**: A structured narrative review of experimental, translational, and clinical studies published between 2010 and 2025 was conducted using PubMed and Scopus. Literature addressing adhesive categories, etching strategies, etching techniques, and smear layer characteristics was critically synthesized to identify functional relationships relevant to bonding performance and clinical decision-making. **Results**: The proposed taxonomy classifies materials as conventional, universal, touch-cure primers, self-adhesive/universal, and glass ionomer cements. Bonding strategies are organized as etch-and-rinse, self-etch, pre-etched, and unassisted, while etching techniques are defined as selective or nonselective families encompassing five clinically defined techniques. Incorporating etching technique clarifies the role of smear layer density, the acidity of adhesive materials, and functional monomer reactivity in demineralization and bonding. This structure enhances the understanding and teaching of adhesive concepts and supports evidence-based clinical selection of materials and techniques. **Conclusions**: Integrating etching technique into adhesive classification provides a functional and dynamic framework that unifies material, strategy, and technique. This taxonomy facilitates clinical decision-making and can evolve with future adhesive formulations. Further independent, long-term studies are warranted to validate the proposed combinations of materials and etching procedures.

## 1. Introduction

Historically, the identification of an adhesive system has been closely tied to the agents used to etch dental hard tissues [1]. The accepted classification of adhesive systems explicitly employs etching-related criteria to divide them into two main categories: etch-and-rinse (ER) and self-etch (SE) [2]. The former requires, as an initial step, the application of rinseable phosphoric acid, which demineralizes the tissues and removes the smear layer, thereby preparing them for monomer infiltration [3]. By a different approach, SE systems use acidic functional monomers that, in a single procedure, demineralize, incorporate the smear layer, and simultaneously infiltrate dental hard tissues to form a hybrid layer [4]. Since their inception, both strategies have been applied to enamel and dentin (and occasionally cementum) without selectivity, which—besides being clinically practical—has yielded favorable long-term clinical outcomes for certain adhesive systems [5,6,7,8].

**Figure 1:**
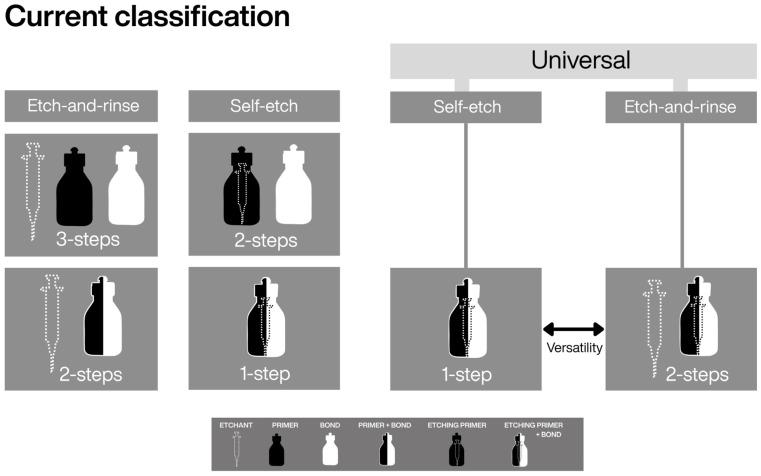
Current classification of adhesive systems, including universal adhesives. The introduction of universal adhesives originated from one-step self-etch systems. The term “universal” identifies the material, whereas the terms “etch-and-rinse” and “self-etch” describe the adhesive strategy. In this hierarchy, strategy refers to the sequence of application steps, without explicit differentiation of the etching technique.

With the introduction of universal adhesives (UAs), this practicality was redefined by offering, within a single system, the possibility of applying both strategies—ER or SE [9,10]. Using the parameters of the current classification, it is evident that under either strategy, UAs exhibit equivalence, at least in their operational steps, to their respective predecessors (Figure 1). However, when a universal adhesive (UA) is used with phosphoric acid, the operator can modulate etching time [11,12] and selectively apply the acid to specific substrates (or avoid it altogether) [13]. This enables deliberate, substrate-specific execution on enamel and dentin, while accounting for smear-layer conditions—variants not explicitly captured by the prevailing step-based classification. Accordingly, etching technique—despite being a determinant of adhesive performance—has been treated mainly as a procedural option rather than as an explicit taxonomic parameter.

In parallel, reformulated and emerging adhesive polymeric materials have appeared, including touch-cure primers, which fall outside conventional adhesive-system classifications [14,15]. In addition, self-adhesive materials are evolving toward universal formulations and are typically classified within cement taxonomies rather than as adhesive options per se [16,17]. Both rely on acidic functional monomers for etching and can be combined with phosphoric-acid pre-etching. These features confer genuine technical versatility. Yet an exclusively step-based classification does not adequately describe these options, complicating interpretation in both academic and clinical contexts.

Within this context, the present work is structured as a narrative review that synthesizes evidence on clinical etching procedures, emerging adhesive materials, and the smear layer as a modulator of bonding mediated by acidic functional monomers. Building upon (and not replacing) the current classification model, a conceptual extension is proposed that explicitly incorporates etching technique as a taxonomic parameter, aligning material type and bonding strategy across adhesive systems, touch-cure primers, self-adhesive/universal materials, and glass ionomer cements. The aim is to provide a clearer and more organized basis for teaching and for evidence-based selection of adhesive approaches.

## 2. Methodological Framework

This narrative review was developed in alignment with general methodological principles commonly applied in scoping reviews, with the aim of enhancing transparency, reproducibility, and systematic organization of the literature, without adopting a formal systematic or scoping review design.

### 2.1. Eligibility Criteria

Eligibility criteria were defined using the Population–Concept–Context (PCC) framework: Population: Studies involving human teeth, dental substrates, or clinical restorative procedures. Concept: Adhesive bonding approaches, including etch-and-rinse, self-etch, self-adhesive, universal adhesives, and touch-cure primers. Context: Experimental, translational, or clinical research evaluating the interaction between etching technique, adhesive category, and smear layer characteristics.

Primary studies addressing the relationship between adhesive materials and etching strategies were considered eligible. Studies evaluating non-commercial or prototype adhesive formulations were excluded.

### 2.2. Information Sources and Search Strategy

Electronic searches were performed from January 2010 to December 2025, in the PubMed and Scopus databases. Additionally, reference lists of included studies were manually screened to identify further relevant literature. No language restrictions were applied.

The search strategy combined controlled vocabulary (MeSH terms) and free-text keywords related to: “dental adhesives”; “etching technique” and “smear layer”. Boolean operators (AND, OR) were applied to maximize sensitivity. The search was first structured for PubMed and subsequently adapted for Scopus.

Foundational and conceptually relevant earlier publications were selectively included when necessary to contextualize the evolution of adhesive strategies and classification models.

### 2.3. Study Selection

All retrieved records were imported into EndNote Web (Clarivate, London, UK), where duplicate removal was performed automatically and verified manually. The selection process involved sequential screening of: Titles; Abstracts and Full texts. Studies meeting the eligibility criteria were included for qualitative synthesis.

### 2.4. Data Extraction and Organization

Extracted data were summarized descriptively and critically synthesized to identify functional relationships among materials, bonding strategies, etching techniques, and substrate conditions, which directly informed the development of the proposed integrative classification framework.

## 3. Etching Techniques Category

Acid etching is the procedural step in which clinically controlled demineralization is mediated by acidic agents applied to dental hard tissues in order to modify their mineral phase and facilitate adhesion. By promoting controlled demineralization of enamel and/or dentin—most commonly through the use of phosphoric acid, or through acidic functional monomers incorporated within adhesive formulations—acid etching regulates interaction with the smear layer and establishes the balance between demineralization and resin infiltration that underlies micromechanical and chemical bonding [1,2].

While acid etching is defined in the current classification as a procedural step within the adhesive sequence, etching techniques describe how this step is clinically executed—namely, through variations in selectivity—thereby influencing the micromechanical and chemical bonding mechanisms of contemporary adhesive materials. Variations in etching selectivity allow the identification and definition of different techniques and, consequently, support considering the etching technique as a functional and taxonomic parameter that complements the step-based classification by facilitating understanding of the clinical application possibilities of a given adhesive material.

**Figure 2:**
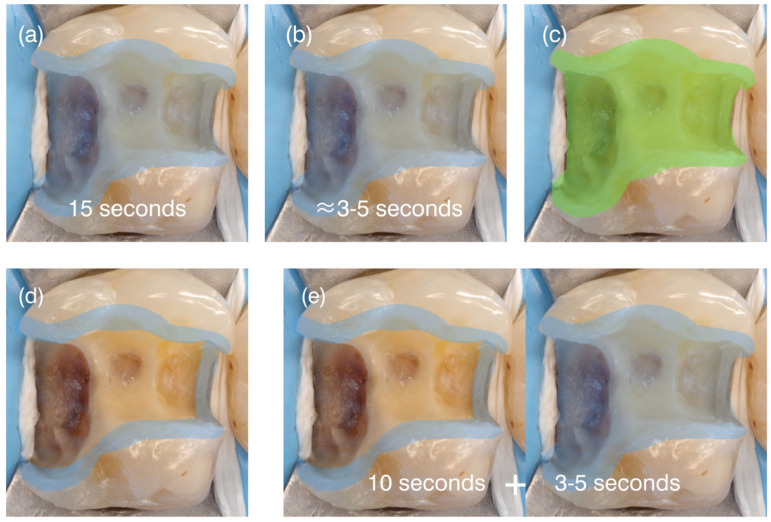
Schematic representation of the four etching techniques within the Etching Techniques Category. Blue areas denote phosphoric-acid application, whereas green areas indicate the action of materials containing functional acidic monomers, which are inherently nonselective toward enamel and dentin. The sequence shows: (**a**) nonselective phosphoric-acid etching; (**b**) short nonselective etching; (**c**) nonselective etching produced by materials with acidic functional monomers; (**d**) selective enamel etching; and (**e**) short dentin etching, in which phosphoric acid is first applied to enamel and then briefly extended to dentin. This figure supports Figure 3 by illustrating how etching techniques function as procedural variables within the proposed framework.

**Figure 3:**
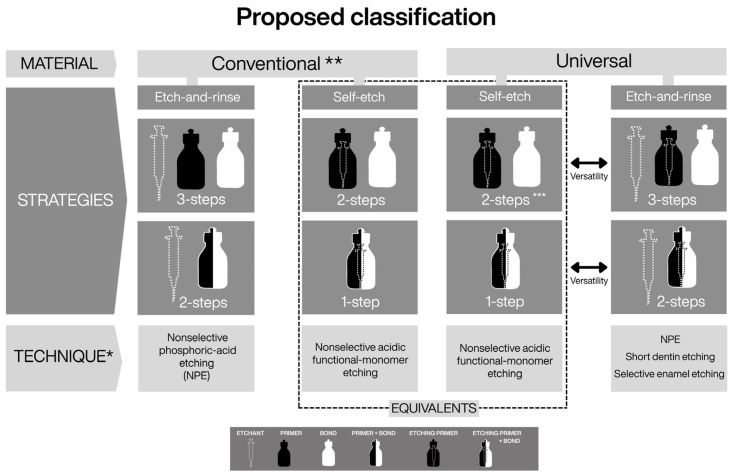
* Incorporation of the “etching technique” parameter into the classification of adhesive systems. ** The term “conventional” is introduced to maintain taxonomic balance with universal adhesives and to distinguish systems that predate their introduction. *** The introduction of two-bottle universal adhesives allows equivalence in the number of procedural steps relative to all preceding systems. Under the proposed framework, functional equivalence is observed between conventional self-etch adhesives and universal adhesives used in self-etch mode (dashed-line box). In contrast, the distinction between conventional and universal etch-and-rinse systems becomes evident only when etching technique is explicitly considered, highlighting clinically relevant differences that are not discernible under the current classification.

Accordingly, within this framework, techniques were classified as nonselective when enamel and dentin are treated identically, and as selective when the substrates are deliberately differentiated. The techniques included in this proposal are described below and illustrated in Figure 2 and Figure 3.

### 3.1. Nonselective Techniques

This group comprises etching approaches in which enamel and dentin undergo identical treatment, without deliberate differentiation during clinical execution.

#### 3.1.1. Nonselective Phosphoric-Acid Etching

This technique typically involves applying phosphoric acid [18,19], at concentrations between 30% and 40%, uniformly over both enamel and dentin for a controlled time, followed by thorough rinsing. The usual application time is 15 s (Figure 2a), although shorter times (≈5 s; “short nonselective etch”—Figure 2b) have been proposed when combined with certain self-adhesive materials [20]. This procedure demineralizes and removes the smear layer, produces optimal enamel microporosities, and exposes the dentinal collagen fibril network [3].

Although effective, the conventional 15 s application increases technique sensitivity and the risk of incomplete dentin hybridization [21], as well as enzymatic degradation of exposed collagen [22]. Under these conditions, adhesion relies primarily on micromechanical interlocking. Within the proposed framework, this technique represents the archetype of a fully demineralizing, nonselective approach.

#### 3.1.2. Nonselective Acidic Functional-Monomer Etching

In this technique, acidic functional monomers incorporated within the adhesive formulation induce etching without a separate rinsing step, contacting enamel and dentin simultaneously [23,24] (Figure 2c). Through this mechanism, localized demineralization, smear layer interaction, and resin infiltration occur concomitantly, resulting in the formation of a hybrid layer that is generally shallower than that produced by phosphoric-acid etching [2].

On enamel, the etching pattern generated by acidic functional monomers is typically less pronounced than that obtained with phosphoric acid; however, it can be enhanced through active application techniques such as scrubbing or sonic agitation [25,26]. In dentin, these monomers act on an initially mineralized substrate, promoting simultaneous demineralization and infiltration while enabling chemical interaction with calcium ions, which contributes to interfacial stability [6,23,24,27].

Because enamel and dentin are treated identically, this approach is classified as a nonselective etching technique within the proposed framework. Its bonding effectiveness results from the combined contribution of micromechanical interlocking and chemical interaction, modulated by the acidity (pH, Pka) and composition of the adhesive material, as well as smear layer characteristics and application dynamics.

### 3.2. Selective Techniques

Selective techniques comprise etching approaches in which enamel and dentin are deliberately treated differently during clinical execution, allowing the operator to modulate both the extent and location of demineralization according to substrate characteristics. In contrast to nonselective approaches, selective techniques explicitly incorporate substrate-dependent clinical decision-making and application control, thereby reducing unnecessary dentin demineralization through selective control of smear layer management and etching extent, while preserving effective enamel conditioning.

By restricting phosphoric-acid application to enamel, selectively modifying dentin etching time, or combining acidic functional monomers with localized phosphoric-acid etching, these techniques optimize the balance between micromechanical interlocking and chemical interaction. As such, selective techniques expand clinical versatility and are particularly relevant when using contemporary adhesive materials with variable acidity and composition.

Within the proposed framework, selective techniques emphasize the role of clinical execution and operator control as key determinants of adhesive performance. The specific selective approaches included in this category are described below and illustrated in Figure 2 and Figure 3.

#### 3.2.1. Selective Enamel Etching

Selective enamel etching involves the targeted application of phosphoric acid exclusively to enamel, while dentin is not subjected to a separate etching step (Figure 2d). In this approach, the smear layer is removed from enamel to generate a retentive microporous surface, whereas on dentin it is preserved and subsequently modified by acidic functional monomers contained within the adhesive material [28].

On enamel, phosphoric-acid etching promotes effective micromechanical interlocking and reliable marginal sealing [29], while also enhancing surface wettability and favoring subsequent chemical interaction between functional acidic monomers and hydroxyapatite through increased MDP–Ca salt formation [30]. On dentin, acidic functional monomers interact with the smear layer, inducing localized demineralization and simultaneous resin infiltration, thereby enabling dentin hybridization while limiting the depth of demineralization [31].

Within the proposed framework, selective enamel etching represents a selective technique based on differential smear layer management and substrate-specific demineralization control. Differentiating the etching approach between enamel and dentin within a single technique aims to enhance bonding predictability when materials incorporating acidic functional monomers capable of effective chemical interaction with dental mineral phases are employed [28,29,31,32,33,34].

#### 3.2.2. Short Dentin Etching

This technique employs different phosphoric-acid application times for enamel and dentin, typically ≈15 s for enamel and 3–5 s for dentin. Clinically, phosphoric acid is first applied to enamel and, after approximately 10 s, extended to dentin for an additional 3–5 s before thorough rinsing [11,35,36] (Figure 2e). An adhesive material containing acidic functional monomers is subsequently applied.

This protocol provides effective micromechanical retention on enamel, ensures smear layer removal on dentin, and limits excessive exposure of the dentinal collagen matrix, thereby preserving residual mineral content that is critical for chemical interaction with the adhesive material [11,36]. Within the proposed framework, this technique exemplifies a selective etching approach based on substrate-specific control of etching time, optimizing the balance between micromechanical and chemical contributions to adhesion.

### 3.3. Summary

In the current classification, acid etching is defined as a procedural step within the adhesive sequence. The present framework complements this view by recognizing that this step can be executed through distinct etching techniques, defined by clinical execution rather than by procedural sequence alone.

By positioning etching technique as a functional and taxonomic parameter, this framework highlights substrate-specific control of demineralization, smear layer management, and application dynamics. Accordingly, nonselective techniques treat enamel and dentin identically, whereas selective techniques deliberately differentiate etching between substrates.

This conceptual organization provides a coherent basis for understanding contemporary adhesive materials—beyond conventional adhesive systems—whose performance depends on both micromechanical interlocking and chemical interaction with dental mineral phases, thereby facilitating teaching and evidence-based clinical decision-making.

## 4. New Adhesive Material Types

The introduction of universal adhesive systems formalized a mode of clinical use that had already begun to emerge with certain self-etch adhesives, in which material formulation allowed selective combination with phosphoric-acid etching at the operator’s discretion [31,32,33]. These developments made explicit the decoupling between material formulation, bonding strategy, and etching technique, identifying technical versatility as a clinically relevant attribute. A prerequisite for such versatility is the intrinsic etching capability of the material, conferred by acidic functional monomers. As this characteristic is no longer restricted to adhesive systems but is increasingly shared by other emerging adhesive resin materials, incorporating etching technique as a taxonomic parameter becomes necessary to coherently interpret their functional potential within an expanded classification framework.

Accordingly, the following sections examine adhesive materials whose physicochemical characteristics and intrinsic etching capability allow them to be coherently interpreted within the proposed classification.

### 4.1. Touch-Cure Primers

Touch-cure primers are hydrophilic, non–light-activated bonding agents—typically containing 10-MDP—characterized by a chemically initiated polymerization that begins upon contact between the primer and the resin composite used for luting [37] or for restorative purposes [14,38]. To achieve this touch-cure reaction, formulations vary among brands and must therefore be paired with the manufacturer-specific cement or restorative resin composite [14].

Compared with their predecessors, these formulations have replaced the conventional benzoyl peroxide–tertiary amine redox system [14,17] with hydroperoxide–thiourea systems [39,40]. In addition, transition-metal salt accelerators (e.g., vanadium(IV) or copper(II)) have been incorporated to control cure rate [39] and increase depth of cure through improved radical diffusion [15,41,42]. With these advances, higher interfacial bond strength is expected [14,41] and mitigation of issues such as color instability [43,44], low degree of conversion in shadowed regions, increased solubility and monomer elution [45], as well as acid–base neutralization between acidic functional monomers and tertiary amines [46,47,48]—a phenomenon reported even when activators were added [49].

Recent results with a touch-cure primer system (Tooth Primer; Kuraray Noritake, Japan) have reported high degrees of conversion and greater bond strength compared with a formulation without accelerators, which would rapidly limit water uptake at the interface [15]. Among the factors associated with stability, the formation of the acid–base resistant zone (ABRZ), located beneath the hybrid layer and generated by the interaction of 10-MDP with dental-tissue hydroxyapatite, is noteworthy [50]. One study showed that dentin interfaces formed with touch-cure primers and subjected to thermal and chemical cycling maintained stable bond strength relative to those obtained with 10-MDP–containing adhesives, with a correlation to ABRZ morphology [51]. This ABRZ has also been successfully described in enamel [52].

**Figure 4:**
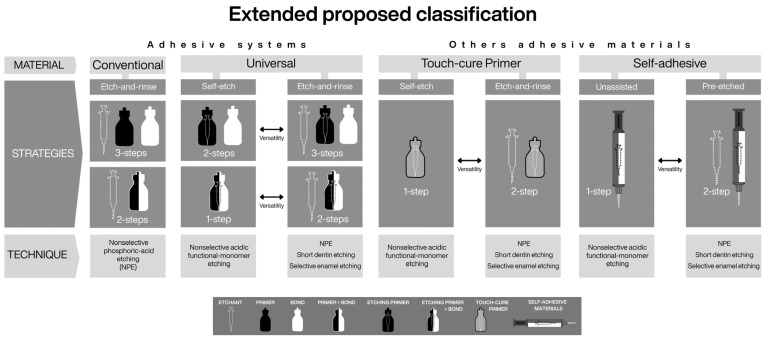
Building upon the classification refinements illustrated in Figure 3, this figure illustrates the versatility of the proposed conceptual framework when applied to other adhesive material categories, showing how material type, bonding strategy, and etching technique can be consistently applied beyond conventional adhesive systems. Conventional self-etch adhesives are omitted, as their bonding strategy and available etching techniques are fully encompassed by universal adhesives used in self-etch mode. The classification retains only universal adhesives and incorporates new material categories—touch-cure primers and self-adhesive materials. Even glass ionomer cements (both conventional and resin-modified) can be interpreted within the self-adhesive material category, as they may rely on nonselective pre-etching with a non-rinse polyacrylic acid.

In fact, the bond strength of touch-cure primer/cement systems may exceed that of newer touch-cure adhesive/cement combinations [37]. Moreover, a recent primer proposal in an auto-polymerizing restorative system (Stela—SDI, Australia) demonstrated superior adhesive capability compared with conventional light-cured resins, with lower risk of pre-failure and fewer voids/gaps in high C-factor cavities [38]. Collectively, this evidence supports considering touch-cure primers as a distinct adhesive material type within the proposed classification. In addition to their distinctive polymerization chemistry, these materials possess intrinsic etching capability through acidic functional monomers, and their clinical performance may depend on the etching technique selected during clinical execution, consistent with the technique-oriented framework (Figure 4).

### 4.2. Self-Adhesive and Universal Resin-Based Materials

The category includes self-adhesive resin composites [2], which by definition are designed to bond to dental hard tissues without the assistance of a separate adhesive system [24]. To achieve autonomous adhesion and simultaneously perform their functional role (e.g., luting), these materials contain acidic functional monomers capable of demineralizing, infiltrating, and chemically interacting with hydroxyapatite [23,24]. Accordingly, when used as originally intended, their etching technique can be considered nonselective, as enamel and dentin are treated identically (Figure 4).

Self-adhesive materials were developed to simplify clinical procedures, reducing both treatment time and operator sensitivity [24]. However, their performance may be limited in low-retention cavity designs, extensively rebuilt preparations [53,54], or enamel-dominant substrates [55,56], thereby restricting their indications. In response to these limitations, the use of phosphoric-acid pre-etching or assistance with adhesive systems has been explored [20,57], with favorable long-term clinical outcomes—up to 15 years—reported when selective pre-etching protocols were employed [58]. In vitro evidence also shows that even short nonselective etching (≈5 s with phosphoric acid applied to enamel and dentin) can improve marginal adaptation and microshear bond strength of self-adhesive cements [20], although compatibility issues between adhesive systems and dual-cure cements have been reported when these materials are used in combination [47,48].

To address these challenges, recent self-adhesive resin-based materials have undergone formulation adjustments that allow combined use with universal adhesives, touch-cure primers, or pre-etching protocols [17]. This evolution—analogous to the transition from self-etch to universal adhesives—has led to the reclassification of some self-adhesive materials as universal cements. These materials typically contain acidic functional monomers such as 10-MDP, 4-MET, or GPDM [17], enabling simultaneous etching and chemical bonding to hydroxyapatite [23], while selected formulations also incorporate silanes to enhance adhesion to silica-based restorative materials [59,60]. Most contemporary universal resin cements are formulated as dual-cure systems incorporating touch-cure technology based on acid-resistant redox initiators. This design ensures adequate polymerization in areas of limited light transmission, where chemical accelerators may further enhance curing in deeper regions [17,41,61]. In addition, polymerization protocols and specific commercial formulations can influence biological performance, with optimized systems demonstrating reduced cytotoxicity and lower biofilm accumulation [16].

Altogether, these developments illustrate a dynamic continuum in adhesion evolution, wherein self-adhesive and universal resin-based materials progressively converge. Their inclusion in this framework reflects the growing need to correlate formulation versatility with clinical outcomes, and to assess how each combination of material, strategy, and etching technique contributes to durable, predictable adhesion.

### 4.3. Summary

The adhesive materials discussed in this section represent the outcome of progressive innovations in composition, polymerization technologies, and clinical procedures aimed at overcoming technical limitations of conventional adhesive systems, simplifying workflows, and improving clinical reliability. These developments justify their consideration as distinct material categories within contemporary adhesive dentistry.

At the same time, despite their specific characteristics, these materials rely on adhesive principles that can be functionally aligned with those governing conventional adhesive systems. By integrating material type, established bonding strategies, and the etching technique as a transversal parameter, the proposed classification provides a coherent framework to interpret their technical versatility and to organize diverse adhesive materials according to shared functional principles.

While material composition and technical versatility influence the application of adhesive approaches, bonding effectiveness is ultimately modulated by the characteristics of the dental substrate, where smear layer management must be considered. Accordingly, the following section examines the role of the dentin smear layer as a key modulator of adhesive behavior.

## 5. Influence of Smear Layer Type on Etching Technique Selection

Correct interpretation of the smear layer—considering the factors that determine its structure and properties—is essential for selecting the most appropriate etching technique for a given adhesive material. The smear layer is a debris film generated during tooth preparation, characterized by a microporous structure that covers the surface and occludes dentinal tubules, thereby reducing dentin permeability [62]. Composed mainly of denatured collagen and hydroxyapatite (HAp) [63], the smear layer can significantly interfere with the performance of adhesive materials containing acidic functional monomers [64,65,66].

**Figure 5:**
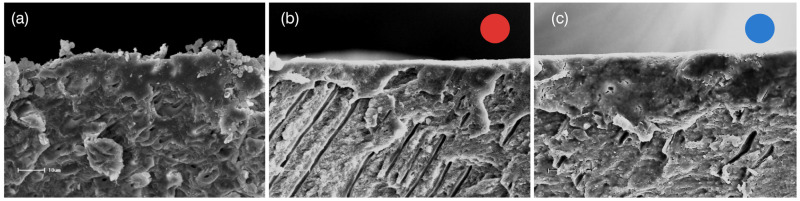
Representative scanning electron microscopy (SEM) images of dentin surfaces demonstrating variations in smear layer morphology according to the preparation method: (**a**) Dentin abraded with SiC sandpaper #600, characterized by a loosely compacted and permeable smear layer, as evidenced by its dispersed appearance; (**b**) Dentin prepared with a fine-grit diamond bur at high speed (red circle), showing a continuous and denser smear layer due to higher condensation; (**c**) Dentin prepared with a medium-grit diamond bur (blue circle), exhibiting a thicker and more compact smear layer compared with the fine-grit preparation.

In clinical practice, diamond and carbide burs are most commonly used for tooth preparation; however, in vitro studies frequently rely on standardized smear layers produced with silicon carbide (SiC) papers—most often 600-grit [67,68]. Because these standardized smear layers are generally less compact and less resistant to dissolution [69,70], such experimental models may underestimate the clinical complexity of adhesive interactions. When diamond burs are employed, both abrasiveness and rotational speed influence smear layer characteristics [71,72]: larger abrasive particle sizes increase smear layer thickness [67,68,73], whereas higher rotational speeds increase compaction and density [68,74,75]. Conversely, low-speed carbide burs tend to produce a thicker but less cohesive and more permeable smear layer [71,72,75]. Among these variables, smear layer density appears to be more critical than thickness in determining the ability of acidic functional monomers to dissolve and penetrate the layer (Figure 5).

The capacity of an adhesive material to dissolve the smear layer and demineralize the underlying tissue is strongly influenced by its acidity [76,77]. Self-etch adhesive systems have been subclassified according to pH as strong (pH < 1), intermediately strong (pH 1–<2), mild (pH 2–2.5), and ultra-mild (pH > 2.5) [4], a classification that can also be applied to universal adhesives. At the time of this review, no strong-pH representatives were identified. Intermediately strong systems promote deeper demineralization and resin infiltration, which may be advantageous on enamel—particularly when combined with active application [25,26]—but may be excessive for dentin [9,10]. From a smear layer perspective, strong and intermediately strong systems are generally not hindered by increased smear layer density or thickness [77,78,79]; however, this does not necessarily translate into superior immediate bond strength [80] or long-term stability [10], outcomes that have often been more favorable with mild and ultra-mild systems [81].

In mild and ultra-mild adhesive systems, acidic functional monomers may be buffered through ionic interaction with the smear layer [64,66,67,74,78,82], reducing their effectiveness in dissolving dense smear layers. Under conditions where the smear layer is more dispersed, hybridization tends to be more uniform [64,69], resulting in higher bond strength [79,82,83]. Consequently, strategies that promote the availability and effective interaction of functional monomers become critical. Two-bottle adhesive systems (primer and bond) are generally recommended [77], while active application, extended scrubbing time, and the application of an additional adhesive layer in simplified systems have demonstrated favorable outcomes [84].

The prioritization of 10-MDP in adhesive formulations has been particularly relevant [85]. Although different acidic functional monomers exhibit variable etching aggressiveness and bonding mechanisms to hydroxyapatite [23]—as described by the adhesion–decalcification concept [1]—10-MDP-containing systems remain the reference standard due to their ability to form insoluble MDP–Ca salts organized in stratified nanolayers and acid–base resistant zones. These interfacial features have been consistently associated with improved bond durability and long-term stability [1].

With respect to self-adhesive materials, demineralization of hydroxyapatite and resin infiltration into dentin may be limited in certain formulations, and these materials often exhibit greater difficulty in dissolving the smear layer when compared with adhesive systems [70,86,87]. While calcium released during hydroxyapatite demineralization may contribute to pH neutralization and improve mechanical properties in some self-adhesive cements [88], lower dentin bond strength and reduced interfacial fracture toughness have also been reported [89,90,91]. On enamel, adhesive efficiency may be further compromised by the presence of aprismatic enamel and smear layer remnants [65]. Consequently, thick and compact smear layers may not be adequately dissolved by self-adhesive materials, impairing hybridization [65,70]. Given that acidic functional monomers differ in their hydroxyapatite-dissolving capacity and calcium-binding potential [23], promoting chemical interaction with dentin by optimizing cement viscosity and surface wetting becomes essential [92]. Accordingly, phosphoric-acid pre-etching has been recommended when using self-adhesive materials [93,94,95], as it improves surface wettability, enhances 10-MDP–hydroxyapatite chemical affinity, and increases MDP–Ca salt formation [30]. Under this rationale, evaluating self-adhesive materials in combination with specific etching techniques—such as reduced etching times on enamel and dentin [20]—becomes particularly relevant within the proposed classification framework.

From a didactic and clinical perspective, classifying smear layers according to the preparation instrument—for example, as favorable (carbide burs; fine or extra-fine diamond burs) or challenging (medium or coarse diamond burs)—facilitates rational selection of etching techniques based on adhesive acidity. In the presence of challenging smear layers, ultra-mild adhesives may benefit from short dentin etching [11], whereas mild adhesives may be more effectively combined with selective enamel etching [81]. For intermediately strong systems, no major limitations in smear layer dissolution are anticipated [96] (Table 1). As emphasized throughout this review, such application optimizations are broadly applicable to adhesive materials containing acidic functional monomers, as they promote more efficient monomer–substrate interaction, enhance chemical bonding and dentinal infiltration, and improve hybridization quality [1,2].

### Summary

The characteristics of the smear layer play a decisive role in modulating the interaction between adhesive materials and dental tissues. By linking smear layer type, the acidity of adhesive materials, and functional monomer chemistry, this section demonstrates that etching technique selection is not merely procedural but represents a rational, substrate-driven adjustment. Within the proposed classification, considering the smear layer as a functional variable reinforces the role of etching technique as a key parameter for understanding adhesive performance and clinical decision-making.

## 6. Conceptual Integration and Functional Rationale of the Proposed Framework

### 6.1. From Step-Based Classification to Functional Versatility

A well-structured classification is essential for translating the complexity of adhesive dentistry into clinically meaningful decisions. The current classification of adhesive systems has been highly successful because it linked material identity to a defined sequence and number of application steps, integrating bonding strategy (etch-and-rinse or self-etch) with procedural execution [3,4]. However, the evolution of adhesive materials—particularly the emergence of universal systems capable of multiple application approaches—has progressively challenged the ability of this framework to fully describe their functional diversity [1,2,17].

Importantly, refining a classification does not diminish the value of existing systems, but rather acknowledges their foundational role while addressing conceptual gaps introduced by material innovation [2,17]. Within this context, the present framework does not replace the current classification, but extends it by explicitly incorporating the etching technique as a functional parameter. This addition preserves taxonomic order while enabling differentiation among materials that share similar bonding strategies but differ in their clinically relevant modes of execution. (Figure 1, Figure 2 and Figure 3)

### 6.2. Etching Technique as the Link Between Material, Strategy, and Substrate

Historically, the terms etch-and-rinse and self-etch simultaneously denoted material type, bonding strategy, and a single, fixed etching technique [3,4]. With the introduction of universal adhesives, these terms now describe application modes rather than material identity, revealing a conceptual shift already implicit in earlier self-etch systems [31,32,33]. The proposed classification formalizes this distinction by separating strategy from technique, thereby clarifying that the number or sequence of steps is not equivalent to how etching is clinically executed.

By specifying the range of possible etching techniques associated with each strategy (Figure 2 and Figure 3), the framework makes evident technical distinctions—particularly between conventional and universal adhesive systems—that are not discernible under the current classification but hold direct clinical relevance [1,81]. In this sense, etching technique functions as a unifying parameter that connects material properties with substrate condition, allowing adhesive behavior to be interpreted beyond procedural labels alone.

### 6.3. Integration of Emerging Adhesive Materials Within a Unified Taxonomy

**Figure 6:**
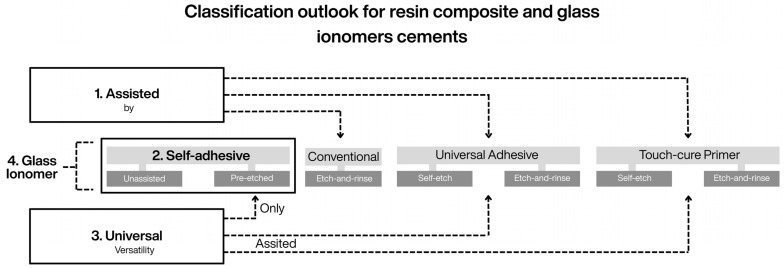
Schematic representation of the influence of the proposed adhesive framework on the functional classification of resin cements, highlighting the relationships among material categories and their modes of association with other adhesive materials. (1) Assisted materials require the use of an independent adhesive agent (adhesive systems or touch-cure primers) as part of the bonding protocol. (2) Self-adhesive materials exhibit intrinsic bonding capability to dental tissues and may be complemented by selective or nonselective pre-etching techniques. (3) Universal cements combine both approaches and may be used independently, assisted with adhesive agents, or in combination with specific etching techniques. (4) Glass ionomer cements (conventional and resin-modified) are also represented, as they function as self-adhesive materials and may involve non-rinse polyacrylic acid pre-conditioning rather than phosphoric-acid etching.

**Figure 7:**
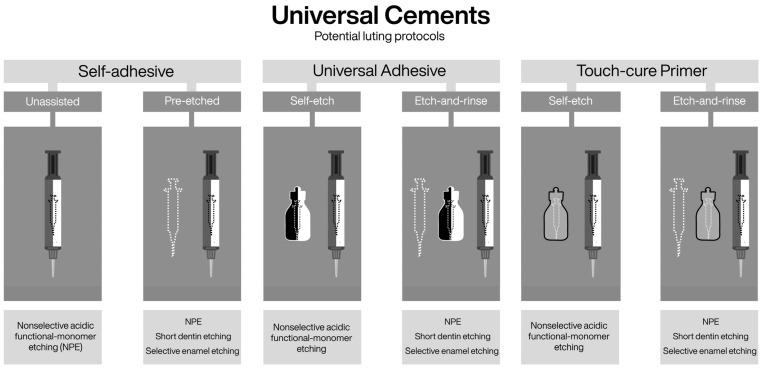
Schematic illustration of the possible clinical associations between universal resin cements and adhesive agents within the parameters of the proposed framework. The figure highlights the multiple application protocols available for these materials, including their use alone, in combination with adhesive systems or touch-cure primers, and with selective or nonselective etching techniques. The incorporation of touch-cure and silane technologies exemplifies the current concept of adhesive universality, in which material compatibility and technique selection determine clinical versatility rather than a single predefined bonding protocol.

The versatility of the proposed framework extends beyond adhesive systems to encompass other adhesive polymeric materials characterized by an intrinsic capacity to etch, infiltrate, and establish micromechanical and chemical bonding with dental hard tissues. Touch-cure primers, for example, can be coherently integrated based on their acidic functional monomer content and their capacity to interact with dental tissues either independently or in combination with phosphoric acid [38,52]. Within this framework, etch-and-rinse and self-etch describe their bonding strategies, whereas etching techniques define their clinical application possibilities (Figure 4, Figure 6 and Figure 7).

Along the same conceptual line, in the proposed framework, the inclusion of self-adhesive materials as an explicit material category establishes a conceptual intersection with resin cement classification [17]. The term self-adhesive is defined here as a functional material category characterized by intrinsic autonomous bonding capability. This conceptual alignment allows self-adhesive resin cements [17], self-adhesive restorative materials [70], and even conventional and resin-modified glass ionomer cements [2] to be interpreted within a common adhesive framework, thereby preserving coherence across taxonomies (Figure 6 and Figure 7)

In addition, the proposed classification contributes to a functional reinterpretation of resin cement taxonomy as a whole. As previously reported [17], resin cements have been classified as adhesive/multi-step materials when used in association with adhesive systems—without explicitly considering the use of touch-cure primers—or as self-adhesive, one-step materials. However, the present framework makes explicit the possibility of combining self-adhesive materials with phosphoric-acid pre-etching, thereby expanding their application beyond a strictly single-step approach, and accommodates universal cements that integrate multiple bonding options.

In addition, the proposed classification contributes to a functional reinterpretation of resin cement taxonomy as a whole. As previously reported [17], resin cements have been classified into three main categories: adhesive/multi-step materials, which require association with adhesive systems; self-adhesive, one-step materials with intrinsic bonding capability; and universal cements, which combine features of both approaches. However, this classification does not explicitly consider the use of touch-cure primers as independent bonding agents, nor does it fully address the possibility of combining self-adhesive materials with phosphoric-acid pre-etching. The present framework makes these possibilities explicit, thereby expanding the functional interpretation of self-adhesive materials beyond a strictly one-step approach and providing a structured rationale for understanding universal cements as materials capable of integrating an extended range of adhesive associations and etching techniques.

Accordingly, building upon this functional reinterpretation, the following conceptual refinements to resin cement classification are proposed: assisted materials, which require a separate adhesive agent (either an adhesive system [17] or a touch-cure primer [14,15,52]) and lack intrinsic self-adhesive properties; self-adhesive materials with intrinsic bonding capability [2,24], whose performance may be complemented by pre-etching following selective or nonselective etching techniques [20,57,58,97,98]; and universal materials, which combine both strategies and may incorporate additional technologies such as silane [17,37]. This functional categorization complements existing cement classifications by adding clinically relevant information related to etching technique selection, material compatibility, and substrate interaction (Figure 6 and Figure 7).

### 6.4. Implications and Future Research Directions

Within the proposed framework, adhesive universality is best understood not as a single material solution, but as the ability to combine compatible materials and complementary etching techniques—applied selectively or nonselectively—to optimize micromechanical retention and chemical interaction according to the substrate and smear layer. Consequently, the concept of “absolute universality” remains an aspirational goal, dependent on further advances in material formulation, chemical compatibility, and long-term clinical validation.

From a research perspective, this framework enables the formulation of clinically relevant questions that cannot be adequately addressed within traditional classifications. These include whether different etching techniques influence long-term adhesion when the same versatile material is used, how compositional variability among touch-cure primers and self-adhesive/universal materials modulates their response to etching techniques, and to what extent smear layer characteristics act as primary determinants of adhesive performance.

## 7. Conclusions

Integrating the etching technique into adhesive classification clarifies the functional relationship among material type, bonding strategy, and clinical execution, providing a coherent framework that complements existing classifications and supports evidence-based decision-making.

Current evidence indicates that smear layer characteristics, the acidity of adhesive materials, and the nature of acidic functional monomers modulate demineralization, resin infiltration, and chemical interaction, and therefore should guide the selection and execution of etching techniques.

The proposed taxonomy coherently accommodates touch-cure primers, self-adhesive, and universal materials, while maintaining consistency with resin cement classifications and extending to self-adhesive restorative materials and glass ionomer cements, based on their bonding behavior and corresponding pre-etching approaches.

Within this framework, contemporary adhesive universality is understood not as a single material property, but as the capacity to combine compatible materials and complementary etching techniques—applied selectively or nonselectively—to optimize micromechanical retention and chemical bonding according to substrate conditions.

Further independent and long-term clinical studies are required to validate the clinical relevance of this functional classification and to refine its application in the context of emerging adhesive materials and evolving formulations.

## Figures and Tables

**Table 1:** Decision matrix illustrating the distribution of etching techniques based on the interaction between smear layer characteristics and adhesive material acidity. The table summarizes recommended etching techniques according to the cross-classification of smear layer type—categorized as favorable (dispersed, permeable) or challenging (dense, compact)—and the acidity of adhesive materials, expressed as ultramild, mild, or intermediately strong pH. The suggested etching techniques presented at each intersection are derived from the available experimental and clinical evidence and are intended to support a didactic, substrate-oriented interpretation of adhesive behavior. As such, this matrix should be understood as a dynamic and evolving tool, whose generalization and refinement will depend on the emergence of higher-level evidence and long-term clinical validation. Within the current state of knowledge, it provides a practical framework linking smear layer morphology, material chemistry, and clinical execution.

	**MATERIAL pH**						**CONVENTIONAL** **Etch-and-rinse**
**SMEAR LAYER**	**Ultramild**		**Mild**		**Intermediary strong**		
	Selective enamel etching		Selective enamel etching		Selective enamel etching Nonselective acid monomer etching		
**Favorable**							Nonselective phosphoric-acid etching
**Challenging**	Short dentin etching Short nonselective etching		Short dentin etching		Selective enamel etching		

## Data Availability

No new data were created or analyzed in this study.

## Abbreviations

The following abbreviations are used in this manuscript:

ER	Etch-and-rinse
SE	Self-etch
NPE	Nonselective phosphoric-acid etching
UA	Universal Adhesive
MDP	10-Methacryloyloxydecyl dihydrogen phosphate
ABRZ	Acid–base resistant zone
